# A systematic literature review of patient perspectives of barriers and facilitators to access, adherence, stigma, and persistence to treatment for substance use disorder

**DOI:** 10.1016/j.rcsop.2021.100029

**Published:** 2021-06-04

**Authors:** Alina Cernasev, Kenneth C. Hohmeier, Kelsey Frederick, Hilary Jasmin, Justin Gatwood

**Affiliations:** a Department of Clinical Pharmacy and Translational Science, University of Tennessee Health Sciences Center, Nashville, TN, USA; b Health Sciences Library, University of Tennessee Health Science Center, Memphis, TN, USA

**Keywords:** Systematic literature review, Opioid use disorder, Methadone, Buprenorphine, Patient, Qualitative

## Abstract

**Introduction:**

The opioid crisis has left a devastating impact on the United States (U.S.) for over 20 years. The over-prescribing of opioid medications and availability of illicit opioids have contributed to the U.S. opioid epidemic. Given the complexity of the epidemic, substance use disorder, and its treatment, there is an urgent need for a thorough review of the qualitative literature which has captured the patient's experiences Such patient-derived qualitative data on lived experiences and perspectives may allow researchers, clinicians, and policy makers to glean new insights into addressing this epidemic.

**Objectives:**

The objective of this paper is to present a systematic literature review of the existing U.S. qualitative research and provide a patient perspective on medications for opioid use disorder (MOUD), including barriers and facilitators to MOUD use.

**Methods:**

In November 2019, four electronic databases (PubMed, CINAHL, Scopus, and Web of Science) were searched by a medical librarian using a combination of keywords, Medical Subject Headings (MeSH), and/or CINAHL subject headings. 8766 results were imported into EndNote, then duplicate records were removed, leaving a total of 4722 articles. The unique records were imported into Rayyan QCRI an online platform designed to expedite screening. Blinded screening was undertaken in duplicate by four reviewers. Two researchers abstracted all the articles and used thematic analysis.

**Results:**

The screening in the abstract phase excluded 4681 results, leaving 41 studies for full-text screening to determine their eligibility for inclusion in the review. After screening, 21 articles were included in the study and the analysis is based on these articles Common themes across studies included stigmatization, perceived barriers to MOUD, and MOUD treatment deserts and provider shortages.

**Conclusions:**

Qualitative research studies conducted to date have uncovered substantial MOUD treatment barriers which are both social and structural in nature. Such barriers to treatment may serve to exacerbate the current epidemic and must be taken into consideration in designing policy and treatment solutions for patients with OUD.

## 1 Introduction

The opioid crisis has left a devastating impact on the United States (U.S.) for over 20 years. In the last decade, the U.S. has shown drastic increases in total opioid overdose deaths, averaging 130 opioid-related deaths per day as of 2018.^1^ Factors contributing to this opioid use disorder (OUD) epidemic include the over-prescribing of opioid medications, development of new opioid extended-release formulations, and availability of illicit substances use including heroin and illicitly manufactured fentanyl and other synthetic opioids.1., 2, 3 Opioid prescriptions become distributed among illegal channels through a process known as diversion.^4^^,^^5^ Approximately 21 to 29% of individuals who have been prescribed opioids misuse them.^5^^,^^6^ It's also been shown that roughly 4 to 6% of people who misuse prescription opioids will go on to use illicit opiates like heroin, and approximately 75% of new heroin users first misused a prescription opioid.^7^, ^8^, ^9^, 10. Together, both prescription and illicit opioids have contributed to the nation's burdensome opioid epidemic.

Several health initiatives have been implemented across the U.S. in effort to address the opioid crisis, reduce over-prescribing of opioids, and support individuals in need of treatment. These include the opioid prescription drug monitoring program (PDMP), opioid stewardship programs, drug take-back events, increased naloxone access, and medications for opioid use disorder (MOUD).^5^^,^^11^^,^^12^ Many states across the U.S. allow the legal dispensing of naloxone without a prescription via a standing order, which has contributed to increased naloxone access and reduced opioid-related deaths (CDC, 2019). Though progress has been made, efforts should continue to further scale naloxone dispensing and access across the country and especially in rural areas.^13^

MOUD, including methadone and buprenorphine maintenance therapy, is an evidence-based treatment modality for OUD demonstrated to reduce illicit opioid use and opioid-related deaths.^14^ However, previous studies have reported a large number of patient-identified barriers to MOUD use, including cost, access, dosing regimens, perceived effectiveness, and associated social stigma.^15^, ^16^, ^17^, ^18^, ^19^, ^20^, ^21^ For example, a leading adherence barrier for patients receiving methadone-based MOUD is the requirement for daily clinic visits required by federal regulations.^17^^,^^22^ According to federal law, methadone may only be dispensed by a certified opioid treatment program. Patients must receive treatment under the supervision of a healthcare practitioner until stable progress and compliance is achieved, at which point patients would be allowed take the medication on their own between appointments.^23^

Underlying many of the barriers to MOUD is the stigma associated with OUD and MOUD, which itself has been linked to worsening health outcomes.^24^, ^25^, ^26^This discriminative stereotyping heavily influences how society views OUD and, resultingly, how individuals with OUD perceive themselves, a term known as internalized stigma.^26^, ^27^, ^28^, ^29^ Internalized stigma itself has been shown to reduce individual's desire to seek initial help for OUD, and over time sustained barriers to engagement in MOUD therapy programs can lead to relapse.^25^^,^^30^^,^^31^ In addition to general impact public stigma, OUD-related stigma has also been studied within healthcare professionals and leads to a variety of negative outcomes. Past negative stigmatizing experiences have caused many patients with OUD to be distrustful of providers and to forego necessary treatment.^32^

Patient perspectives on OUD and MOUD are critical to understanding both treatment goals and barriers to access. Yet, because of the associated stigma, these perspectives are generally hidden from key decision makers like medical professionals and policy makers. Published systematic literature reviews to date have focused on safety and efficacy data of OUD treatment options and varying dosages.^33^, ^34^, ^35^ Currently there are no systematic literature reviews that explore patient perspectives of MOUD. Such a review of the literature focused on understanding individuals' perspectives and lived experiences with OUD and MOUD would be advantageous for both patients and providers, enabling the provision of more individualized, quality patient care. This includes obtaining a better understanding of patient-perceived facilitators and barriers towards treatment, adherence, access, and the impact of social stigma. By learning and understanding patients' perspectives, improvements can be made in care, adherence, and outcomes and contribute towards our fight against the opioid epidemic. Given the lack of previous systematic reviews regarding patients' perceptions of OUD treatment, the present study set out to explore and evaluate existing U.S. literature in this area. Greater insight into the perspectives of individuals with OUD (including perceived barriers and facilitators to buprenorphine and methadone treatment, access, and adherence) is needed to better understand and predict patients' experiences, motivators, and outcomes and to begin to rid the world of OUD- and MOUD-associated stigma. The objective of this paper is to present a systematic literature review of patient perspectives on MOUD, including barriers and facilitators to MOUD use.

## 2 Methods

This systematic review was undertaken to identify qualitative and mixed method studies that reported on patient perspectives of barriers and facilitators to access, adherence, and persistence to buprenorphine/methadone treatment for Opioid Use Disorder (OUD). This review was conducted in compliance with the Preferred Reporting Items for Systematic reviews and Meta-Analyses (PRISMA).

In November 2019, four electronic databases (PubMed, CINAHL, Scopus, and Web of Science) were searched by a medical librarian using a combination of keywords, Medical Subject Headings (MeSH), and/or CINAHL subject headings. Grey literature was not explored due to a limited amount of literature and to limitation on study design criteria. The final search strategy can be found in the Appendix 1.

8766 results were imported into EndNote, version 9 (Clarivate, Philadelphia, PA), then duplicate records were removed, leaving a total of 4722 articles. The unique records were imported into Rayyan QCRI (Qatar Computing Research Institute, Doha, Qatar), an online platform designed to expedite screening. Blinded screening was undertaken in duplicate by four reviewers. Studies were limited to the English language, and to publication dates 2000–2020. The titles and abstracts were retrieved, imported into Ryyan and reviewed blindly by three authors. The fourth author served as an arbitrary role.

### 2.1 Inclusion and exclusion criteria

The reviewers used the inclusion and exclusion criteria that was developed by consensus. All abstracts that were not social behavior research-based were excluded. All articles that were based on an exclusively quantitative method were excluded such as randomized controlled trials, cohort and case studies, epidemiological studies, knowledge, attitudes and practices studies conducted outside the US, studies conducted during pregnancy, studies conducted with an incarcerated population, and other studies using only standardized questionnaires. Furthermore, articles that used mixed methods, such as a mixture of standardized questionnaires, self-report questionnaires, and focus-group discussions and/or participant observation, were examined to determine whether they were predominantly quantitative or qualitative by nature. Those articles that used primarily quantitative methods were excluded.

### 2.2 Screening of studies

The screening in the abstract phase excluded 4681 results, leaving 41 studies for full-text screening to determine their eligibility for inclusion in the review. In screening the full-text, 20 articles did not meet criteria and were excluded. This left 21 articles that qualified for inclusion in the review. These 21 articles were based on qualitative research conducted in the US, focused on issues surrounding OUD, and were written in English. The rational for conducting a systematic literature review on qualitative studies is that qualitative studies mainly aim at providing in-depth and nuanced data on the social, behavioral, and cultural aspects of OUD. On the contrary, quantitative studies focus mainly on common trends across a population.

## 3 Results

A total of 21 studies were included in this narrative review. The first two authors abstracted the articles that contained the following categories: authors, year of publication, source, title of publication, period of study, population demographics (e.g. gender, age, place of residence of informants), number of qualitative interviews, number of focus groups, main research question, main results, and recommendations.^36^^,^^37^ The information extracted from each study was entered into a table to provide an overview for a thematic analysis of the articles. Furthermore, both authors (AC, KH) reviewed each article for content analysis and each article has been summarized.^37^ The authors met to discuss and reached consensus for the thematic analysis.^38^

In the same time, both authors also assessed the risk of bias using CerQual Evaluation. The GRADE CERQual Evaluation assessed the following criteria Methodological Limitations; Relevance; Coherence and Adequacy of data. Using GRADE CERQual criteria, the team met and discussed the studies and used recommendations such as high, medium, and low.^39^ Out of 21 studies, 16 were evaluated as high confidence, two received medium confidence, and three fell into low confidence GRADE. Finally, the articles have been subject to a content analysis where the textual information in each has been summarized.

The thematic analysis of these 21 qualitative studies, the following main points were extracted. Table 1 highlights the main points for each study.

**Fig. 1:**
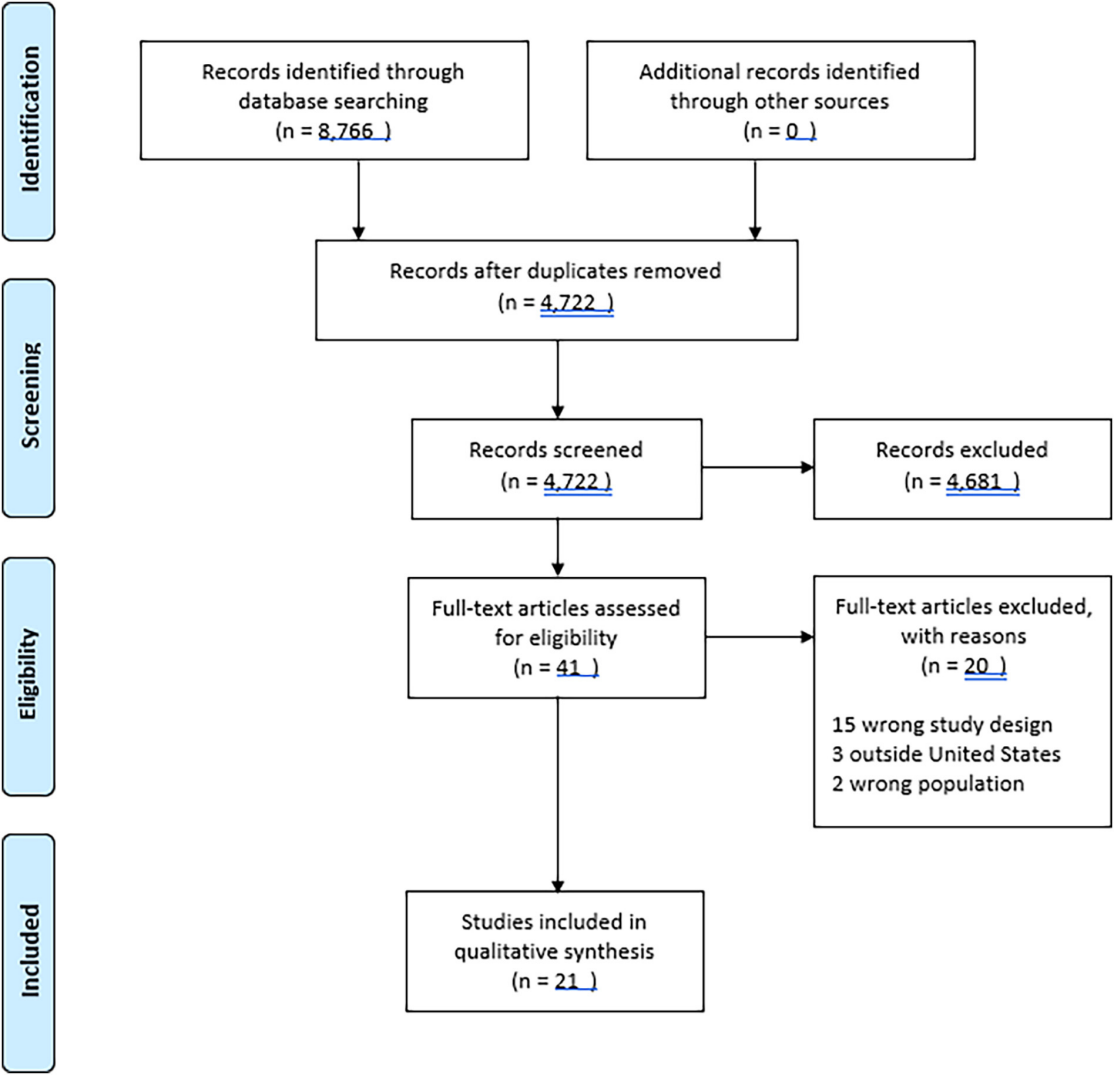
PRISMA flow diagram.

• None of these studies included observation or participant observation in their data collection.
• Studies were conducted in different geographic areas of the US and showed diversity.
• Out of 21 studies, 17 studies used mainly semi-structure interviews in data collection, the number of participants varying from 11 to 77.
• None of these studies included observation or participant observation in their data collection.
• The majority of studies used a single method of data collection.Out of the total of 21 studies, 1 used mainly focus groups
• Three studies combined focus groups and interviews.
• One study focused on barriers such as external health plan policies on formulary coverage, benefit management, reimbursement, the lack of physician linkages to use extended release naltrexone.
• A number of studies focused on the barriers from the patients' perspective on methadone treatment waiting lists, lack of money, lack of health insurance or any insurance issues that could prevent them from accessing methadone center, and transportation.
• Stigma is an important obstacle to MOUD pausing delays in care.
• A number of studies explored the issues related to patient-centered care access in MOUD and highlighted various obstacles to retention with OUD care. (See Fig. 1.)

**Table 1:** Study summary for systematic literature review.

Article and year	Methods: Interview (I) Focus Group (FG) Both	Barriers to care	Stigma	Physician role in patient care	Participants experiences	Pharmacist role: naloxone; patient engagement	Geographic Area
Alanis-Hirsh et al.*,* 2016	I	*			*		Oregon, California, Wisconsin
Bailey et al.*,* 2014	I					*	Massachusetts, Pennsylvania, Washington
Bearnot et al.*,* 2019	I		*				Not specified
Chatterjee et al., 2018	I				*		Massachusetts
Fleming et. al 2019	FG					*	Texas
Fox et al.*,* 2015	I	*	*		*		New York
Furst et al*,* 2013	I	*	*	*	*		New York
Godersky et al, 2019	Both	*	*	*	*		Northwest US
Gunn et al., 2016	Both		*		*		New York
Hatcher et al.*,* 2018	I		*		*		Not specified
Heinz et al.*,* 2010	I	*	*		*		Maryland
Hewell et al.*,* 2017	Both	*		*	*		Alaska
Kang et al., 2019	I			*		*	North Carolina
Mathis et al.*,* 2020	I				*		Tennessee
Mitchell et al.*,* 2011	I	*		*	*		Not specified
Peterson et al.*,* 2010	I	*					Maryland
Teruya et al., 2014	I	*		*	*		Not specified
Tofighi et al.*,* 2019	I	*			*		New York
Velez et al.*,* 2017	I			*			Oregon
Wilson et al., 2018	I	*			*		Pacific Northwest
Yarborough et al.*,* 2016	I	*	*				California

### 3.1 Participants perspective on roots of stigma in OUD access and treatment

Stigmatization in general, the role of perceived and enacted stigma, and its consequences on MOUD were presented in these studies. The studies which address issues of contributing factors to stigma focus on how the complex relationship between knowledge and social conditions, such as homelessness inform an individual's response to stigma. Three articles address the issue of stigma directly, while the other studies address stigma while as a component of barriers to treatment access.

Gunn et al. highlighted the complex interplay between policy and societal norms in creating social stigma, and how these factors deters those with OUD from seeking care. The study was conducted with Russian immigrants living in the U.S. and uncovered the stigmatizing attitudes felt by individuals with OUD from their immigrant community.^40^ Interestingly, it was noted that the Russian immigrant community was more accepting, and consequently holds fewer stigmatizing views, towards heavy drinking. This dichotomy was believed to have stemmed from societal norms within Russian culture, which was influenced by past Soviet Union policies which penalized drug-related addiction, while largely normalizing alcohol-related addiction.^40^ Beyond this stigma towards OUD serving as a barrier for individuals to seek MOUD, it also was found to reduce parental monitoring of early signs of OUD in children until the OUD was severe.

In Bearnot's study, the role of stigma and the discrimination as a barrier to OUD-related care was emphasized by both OUD patients and providers.^41^ Participants noted delays in overall care, including limited home care options, patients with OUD faced – explaining that these individuals were singled out as “second class citizens” by healthcare providers when seeking care for both OUD and OUD-related diseases (i.e. endocarditis).^41^ Other participants were made to feel as if they were a waste of care resources, noting that physicians and nurses would use terms like “this is your fault” and “we're not going to do it twice” when referring to corrective surgical care.

A number of studies focus on stigma from the perspective of treatment seeking behavior.^42^^,^^43^ The main conclusion from the Hewell et al. study is that participants' beliefs played an essential role in the treatment-seeking process, and that intrinsic motivators for long term recovery were dependent on “will, dedication, clarity of values, and spirituality.” Participants felt that others had negative views patients on MOUD because they were still “addicts,” and not instead in the recovery process. These negative social views also impacted the participants' continued progress in the treatment-seeking.^43^ Similarly, Hatcher et al. uncovered the differing social needs of their participants' based on social-economic status and ethnicity.^42^ The article concludes that an individualized understanding of the “multiple oppressions and survival needs” of peoples with OUD is a necessary prerequisite to improved MOUD adherence and treatment seeking. Chatterjee et al. also highlighted stigma as an obstacle to seeking care for participants not enrolled in the OUD treatment.^29^

### 3.2 Lived experiences of patients from initial entry into MOUD to continued recovery

The lived experiences of participants' as they undergo methadone and buprenorphine MOUD treatment is discussed. The moderating roles of personal relationships and spirituality in participants' recovery are taken up in many articles. Furst (2013) and Godersky (2019) found that non-adherence to buprenorphine was due to various characteristics, including social, structural, and logistical aspects (e.g., transportation, homelessness, forgetfulness). Participants indicated that intentional non-adherence occurs intermittently so as to allow for occasional substance use or diversion. For example, Furst et al. findings indicate that the participants use buprenorphine/naloxone to manage withdraw between periods of heroin use.^44^ Godersky's results further highlight participants' participation in diversion, refraining from taking medication to use illicit drugs, forgetting to take medication, and splitting doses. This study shows that the participants perceived video directly observed therapy (VDOT) as an opportunity to increase trust from providers concerned about diversion and responsibility.^19^

Furthermore, Hatcher et al. show the differences between races and social-economic class plays a significant role in treatment access. For instance, low-income African Americans and Latinos individuals faced office-based buprenorphine treatment as isolating.^42^ On the contrary, educated Caucasians have a better understanding of treatment access and are resourceful for seeking support from outside of the clinic.^42^ In the same vein, Toffigi et al.'s also report that participants limited access to programs in less affluent geographic areas played a significant role in seeking MOUD treatment.^45^ Although some participants in this study were interested on starting treatment with buprenorphine, they expressed limited familiarity with extended-release naltrexone.^45^

An article by Wilson et al. put forth a new theory positing that for those patients with chronic pain a pathway exists from addiction to MOUD. The participants experienced a perceived symbiotic relationship between physical and mental pain (e.g., anxiety, depression, stressors), including the constant search to obtain relief by using opioids to an eventual outcome of participating in a MOUD.^46^ Wilson further posits that relationships are a moderating variable with can both serve as a barrier and facilitator to MOUD treatment.

In several studies focusing on methadone treatment, common subthemes include how social expectations influence the participant's decision to be part of the treatment, discordance between patient and provider MOUD treatment goals, and reasons for treatment seeking motivations. In Mitchell's study, the participants were interviewed when they started the methadone treatment, then at four, eight- and 12-months post-treatment entry. This study indicates that there is variability regarding the participants' goals and their reasons for seeking treatment. The authors also highlighted that failure “to abide by treatment clinic rules do not necessary constitute ‘treatment failure’ from the perspective of patients, who often wish to remain in treatment even if it is not progressing optimally from the program's perspective”.^47^ Furthermore, the study indicated that MOUD treatment motivation was dependent on “a critical event or being at a stage of life that prompted emotional and psychological growth.” Peterson et al. illustrate how treatment access barriers including economic burden of treatment (i.e. lack of financial means or health insurance) and lack of transportation, leaves the participant with poor treatment outcomes. Furthermore, participants' beliefs, primarily influenced by their peers, about methadone's possible side effects and reservations about the maintenance program made them unwilling to enroll in the methadone program.^48^

One study encompassed eight community-based opioid treatment programs across the U.S. and the participants were enrolled up to three-and-a-half years after participating in a randomized clinical trial comparing the effect of buprenorphine/naloxone and methadone.^49^ The interviewed participants described the barriers and facilitators to buprenorphine/naloxone or methadone. The study combined both general and study-specific barriers and facilitators. General MOUD treatment barriers included a first-hand negative experience with MOUD, access (e.g., transportation, distance to clinic), and competing priorities for the patient. General MOUD facilitators included first-hand positive experience with MOUD, prioritizing MOUD treatment, MOUD staff support. Although mention was made of many patients preference for methadone over buprenorphine/naloxone, there was no deeper exploration of this phenomenon in the study.^49^

### 3.3 MOUD treatment deserts and provider shortage

This theme addressed specifically the structural barrier of MOUD treatment deserts and MOUD provider shortage. For example, Chatterjee described care obstacles including transportation issues, fluctuations in treatment locations, and shelter assignment changes to access treatment.^29^ These location-specific barriers to care access were sometimes referred to as “distance to treatment,” “gasoline cost,” or “competing priorities,” among other descriptors – but all centered around a lack of convenient access to MOUD treatment centers.

Similarly, several studies discussed the idea of an MOUD provider shortage and legal and regulatory restrictions prohibiting midlevel providers from engaging in MOUD treatment. There was a total of three studies focused on the pharmacists' roles in the opioid epidemic. Bailey et al investigate pharmacists' general awareness and perceptions of how to prevent opioid overdose and their role in the usage of naloxone.^50^ The study concluded that pharmacists are well-positioned to impact the opioid use population by identifying high-risk patients because they have direct access to at-risk individuals to both screen and subsequently provide naloxone. Following the same line, Fleming et al concluded that the community pharmacists are well-positioned to refer participants with OUD to the appropriate treatment and use this opportunity to bring awareness about abuse or misuse of opioids.^51^ The main focus is on the need and opportunities for collaboration between physician and community pharmacists to manage chronic non-cancer pain in the context of the opioid epidemic.^52^ This study discusses the healthcare system's weaknesses that healthcare professionals have to mitigate opioid abuse and diversion.^52^ One of the most critical weaknesses discussed by physicians is the need for additional supports and resources for chronic pain management. At the same time, pharmacists identified cost as the major obstacle to initiate new services.^52^

## 4 Discussion

This systematic literature review's main goal was to provide an in-depth and detailed understanding of the socio-cultural issues that participants with OUD face within the U.S. The studies included in this narrative review show the unfolding complexities of the opioid epidemic at the individual, community levels, and the stigma associated with OUD. Contrary to quantitative research that provides critical information about the studied population's epidemiological patterns, the qualitative studies presented here add rich insights into the participants' lived medication use experiences, treatment goals, and perspective of the opioid epidemic.

It is important to note that many of the studies provide little information about the actual time of data collection. This information is vital to better understand the patient perspectives on MOUD. Furthermore, a limited number of studies discussed how they achieved rigor and trustworthiness in their methodology and did not provide the methodology for this.

One of this systematic literature review's findings is the preponderance of stigma-based studies identified despite some of these studies do not have a stigma as their research objectives. The stigma arose as a theme or a collateral theme, as the researchers listened to the participants' voices.

The stigma associated with the opioid epidemic is crucial. While various studies discussed stigma as a barrier to adherence to MOUD or not initiate MOUD treatment, relatively few studies examined explicitly viable solutions. However, themes uncovered do present a variety of possible avenues from which future research may uncover such solutions.

Mitchell et al., identified discordance between what a provider or MOUD program deemed treatment failure, and what failure meant to the patient seeking treatment. Such discordance also exists between the patient and provider with expectations of the program and overall treatment goals. This perhaps highlights the need to identify “best practices” in communication and MOUD program policies which close this gap in perceived treatment failure, in addition to exploring what treatment goals are of particular value to the patient even if not it's highly prioritized by the provider or the scientific community. There is also needed to better understand intrinsic motivation for treatment seeking and program adherence behaviors. Several of these motivators were identified by the studies included in this review and include the desire to foster and maintain social connections, clarity of values and purpose, and maturity that comes from time and experience. However, the complex interplay between these factors remains elusive and warrants further exploration.

Feelings of being valued less than other patients or persons in society was also a common theme – and although changing public perceptions may be significant challenge, facilitating an environment within the MOUD clinic more conducive to this patient population maybe markedly less so. For instance, a recent qualitative research study in patients with injectable substance use disorder identified that building trust and supportive relationships with treatment center staff what is a critical factor in their treatment success.^53^ This further evidences the findings from the Godersky et al. which pointed to the use of technology as a tool to improve this trust, and subsequently treatment success. Furthermore, research should explore how to destigmatize behavior from the healthcare services point of view or how to incorporate participants opinions to improve the patient-provider or patient-staff experience through training and interventions which target the culture within these treatment centers.

Access barriers to MOUD treatment also present a challenge. They include cost, MOUD treatment deserts, healthcare provider shortages, insurance coverage, and transportation. There is limited literature that shows how many participants are enrolled and retained in the MOUD, although the number is almost certainly well below treatment statistics for less stigmatized chronic disease states. The significant contribution of restrictive program structures, lack of continuity of care, and a culture resistant to the use of medications to treat OUD are key to understanding the limited usage of MOUD within the U.S.^53^ Furthermore, it will be important to garner general perspectives of individuals with OUD in terms of narrative qualitative studies. The uncovering of lived experiences prior to entry into MOUD treatment may serve to facilitate better screening, referral, and public outreach efforts. For instance, a recent study in Tennessee identified that beyond traditional addiction and tolerance pathways to perpetuate OUD, the use of licit and illicit opioids as a revenue source for individuals also serves as a complicating factor in treatment and recovery.^55^

At this particular stage of the opioid epidemic, one can argue that more and robust qualitative research is necessary to better understand the patients' MOUD use experiences. Despite the well documented structural, sociocultural, and individual barriers to treatment, patients seek out and maintain recovery in these opioid treatment programs across the U.S. Understanding the motivations and facilitators of these MOUD-adherent patients may hold the key to better designed care access strategies (e.g., convenient MOUD treatment locations and expanding the provider pool to include other midlevel providers including pharmacists), improved provider-education and training, and public awareness campaigns that make use of the captured lived experiences and stories of this highly stigmatized population.^56^^,^^57^

## 5 Limitations

The current systematic literature review on qualitative studies should be interpreted in light of its limitations.

Firstly, grey literature such as non-peer-reviewed reports, conference abstracts, masters or doctoral thesis, and commentary papers have been excluded. Secondly, there were a limited number of qualitative studies that addressed the role of pharmacists on the US population. Although numerous qualitative studies were conducted globally, the focus of the current systematic literature review was on the US population. Further systematic literature reviews on qualitative studies could compare and contrast the US's findings with global ones. Thirdly, the current systematic literature review could not capture many qualitative studies focused on the pharmacists roles in MOUD. Finally, studies conducted on social media were not included in the review due to the novelty of the field and the lack of tools to assess the bias. This suggests that future systematic literature reviews should focus on social media research that could bring more evidence to this subject.

## 6 Conclusions

This systematic literature review covering US qualitative studies published between 2000 and 2020 identified some common themes such as stigmatization, aspects of the healthcare system regarding MOUD, perceived barriers to MOUD, and pharmacists' uprising role in this population. This systematic review shows that there are certain commonalities across the US.

## Author contributions

A.C., K.C.H., and KF researched literature and conceived the study. A.C., K.C.H., KF, HJ, and JG were involved in protocol development. HJ did the literature search. A.C., K.C.H., KF, JG did the literature review. A.C. and K.C.H. abstracted all the studies. KF wrote the introduction. AC wrote the first draft of the manuscript. All authors reviewed and edited the manuscript and approved the final version of the manuscript.

## Funding

This study was not funded.

## Declaration of Competing Interest

The authors declare that they have no known competing financial interests or personal relationships that could have appeared to influence the work reported in this paper.

The authors declare the following financial interests/personal relationships which may be considered as potential competing interests:

## Appendix A Appendix 1. Algorithm for literature search

(((((((“Opioid-Related Disorders”[Mesh] OR “opioid use disorder” OR “OUD” OR “opioid abuse”)))) AND ((((((((“Methadone”[Mesh]) OR “Buprenorphine”[Mesh]) OR “Analgesics, Opioid”[Mesh]) OR “Opiate Substitution Treatment”[Mesh])) OR ((“opioid agonist therapy” OR “opioid agonist treatment” OR “analgesic” OR “analgesics” OR “opioid” OR “opioids” OR “methadone” OR “buprenorphine”)))))) AND (((((((((“Medication Adherence”[Mesh]) OR (“Treatment Adherence and Compliance”[Mesh])) OR “Treatment Refusal”[Mesh])) OR ((“medication adherence” OR “medication compliance” OR “medication persistence” OR “treatment refusal” OR “adherence” OR “compliance” OR “noncompliance” OR “concordance”)))))) OR (((((((“Patient Preference”[Mesh]) OR “Health Knowledge, Attitudes, Practice”[Mesh] OR “Patient Acceptance of Health care”[Mesh] OR “Social Stigma”[Mesh] OR Shame[Mesh])) OR ((“experience” OR “experiences” OR “perception” OR “perceptions” OR “shame” OR “shames” OR “stigma” OR “stigmas” OR “barrier” OR “barriers” OR “quality of life” OR “attitude” OR “attitudes” OR “belief” OR “beliefs” OR “perspective” OR “perspectives”))))))))))
